# Recent Progress in Hospital Construction

**Published:** 1912-02-17

**Authors:** 


### The Hospital

The Workers' Newspaper of Administrative Medicine and Institutional Life, Administration, National Insurance and Health.

No. 1336, Vol. LI. SATURDAY, FEBRUARY 17th, 1912. PRICE ONE PENNY.

### RECENT PROGRESS IN HOSPITAL CONSTRUCTION.

Nearly every year a number of new hospitals are built or planned, and in the circumstances it is not to be wondered at that every year a number of new features of practical interest in hospital construction are presented for the consideration of the architect, the superintendent, or the surgeon or physician. It is, of course, an undoubted fact that only very rarely does a new hospital revolutionise a point of construction or introduce what may legitimately be claimed as an entirely original idea into hospital planning. What is more often the case is that every new design means the development of an old idea along original lines; an experiment, in other words, which must necessarily possess a great deal of interest to those who follow with attention the progress of hospital work. From time to time we have given an account in these pages of new plans and designs which contain some feature of interest, or which hold out the promise of producing an innovation of practical value in construction. These plans speak for themselves, and it is unnecessary here to detail their special features. It may, however, be instructive briefly to point out the main lines along which progress has chiefly been made during the last year.

To English hospital workers two large institutions now in course of erection, King's College Hospital and the Bradford Infirmary, will appeal in the first place. Both are by the same architect, but they are different in conception and execution, and one, the Bradford institution, presents many interesting features. Perhaps the first point that strikes one on looking over the plans is the comparative grandiose scale on which the designs have been conceived. Seven years ago in Germany and fifteen years ago in America the tendency was in the same direction: the supposition was held that a hospital should be architecturally as beautiful as possible, and expense was made a secondary consideration to architectonic elegance. The result of this tendency is seen in such magnificent buildings as the administrative block of the Moabite Hospital, and the whole of the Mount Sinai Hospital. But a more sober view was not long in displacing this extravagant conception of the essentials of hospital construction. The Continental hospitals, with one or two exceptions, which have been erected during the past year are distinguished by a soberness of exterior which gives dignity without undue display and by a rigid simplicity and an absence of fads which should be the rule in hospital construction. As an illustration we may instance the surgical clinic at Graz, the new hospital at Vienna, the children's hospital at Breslau, and the annexes to the hospitals at Milan, the new buildings of the Bayonne hospital, and the plans for the new Jewish hospital at Berlin. In America the same return to the saner methods of hospital construction is to be observed. The plans for the new Presbyterian hospital are eminently worthy of study, and this institution, when completed, should be one of the finest in the world, although it contains certain features which are not likely to be universally approved of by hospital workers.

Another feature of the past year has been the tendency to revert to the block style instead of favouring the pavilion system, and to make the wards smaller than has hitherto been the rule, consistent with proper supervision. It is also noticeable that the area provided for the ward annexes has steadily grown, so that now it is common enough to meet with a pavilion in which the area of the annexes equals that of the ward itself. Greater simplicity in the furnishing of the wards, the theatres, and the annexes, the use of labour-saving devices wherever possible, and an increased desire to make the institution independent of outside sources, are other points to be noted. In addition attention may be drawn to the fact that larger hospitals are now being built in preference to small institutions, the statistics having conclusively proved that the small hospital is a more expensive and less conveniently administered institution than the large one. Cottage hospitals, of course, are exempt from this generalisation, and during the past year considerable improvements have been made in their designing and equipment. In regard to technical points the most noticeable feature is perhaps the doubtful use of composite tiles for floorage and roofing, the tentative employment of aluminium wall-plates to deaden sound, and the improvements in methods for making walls damp-proof or damp-resisting. New methods of heating and ventilation have not been attempted, but modifications of old methods have been adopted in several new institutions; in the main the tendency in this direction has been to employ central heating and natural ventilation as much as possible.

A great deal of attention has been paid to the special departments, especially the balneo-therapeutic and electro-therapeutic departments. These have been designed on somewhat novel lines at Berlin, Krakow, and Naples. In the surgical department the striking feature has been the general adoption of the high or low pressure cabinet for intra-thoracic operations. One of the best models of this, designed during the past year, is in the new operating room at the German Hospital, New York. It is more and more becoming clear that the special departments need the careful attention of the hospital architect, and that many of the old models hitherto slavishly followed in constructing them are inherently wrong. The laundry and bakery have also received considerable attention, and the plans of new departments, so far as these two branches of hospital work are concerned, present many points of interest. Finally the mortuary has come into its own during the past year. The new mortuaries built have in some cases been equipped with bathrooms and work and retiring rooms, while the arrangement of the chapel and private rooms for friends has been altered. Unfortunately progress in this direction has chiefly been confined to Continental hospitals; in England it is still too often thought that a post-mortem attendant does not need a bath-room and that to expend money and care on improving the mortuary is to err on the side of extravagance.

